# Stabl: sparse and reliable biomarker discovery in predictive modeling of high-dimensional omic data

**DOI:** 10.21203/rs.3.rs-2609859/v1

**Published:** 2023-02-28

**Authors:** Julien Hédou, Ivana Marić, Grégoire Bellan, Jakob Einhaus, Dyani K. Gaudillière, Francois-Xavier Ladant, Franck Verdonk, Ina A. Stelzer, Dorien Feyaerts, Amy S. Tsai, Edward A. Ganio, Maximilian Sabayev, Joshua Gillard, Thomas A. Bonham, Masaki Sato, Maïgane Diop, Martin S. Angst, David Stevenson, Nima Aghaeepour, Andrea Montanari, Brice Gaudillière

**Affiliations:** 1 Department of Anesthesiology, Perioperative & Pain Medicine, Stanford University, Stanford, CA; 2 Department of Pediatrics, Stanford University, Stanford, CA; 3 Télécom Paris, Paris, France; 4 Department of Pathology and Neuropathology, University Hospital and Comprehensive Cancer Center Tübingen, Tübingen, Germany; 5 Division of Plastic and Reconstructive Surgery, Department of Surgery, Stanford University, Stanford, CA; 6 Department of Economics, Harvard University, Cambridge, MA; 7 Sorbonne University, GRC 29, AP-HP, DMU DREAM, Department of Anesthesiology and Intensive Care, Hôpital Saint-Antoine, Assistance Publique-Hôpitaux de Paris; Paris, France; 8 Department of Biomedical Data Science, Stanford University, Stanford, CA; 9 Department of Statistics, Stanford University, Stanford, CA; 10 Department of Electrical Engineering, Stanford University, Stanford, CA

## Abstract

High-content omic technologies coupled with sparsity-promoting regularization methods (SRM) have transformed the biomarker discovery process. However, the translation of computational results into a clinical use-case scenario remains challenging. A rate-limiting step is the rigorous selection of reliable biomarker candidates among a host of biological features included in multivariate models. We propose Stabl, a machine learning framework that unifies the biomarker discovery process with multivariate predictive modeling of clinical outcomes by selecting a sparse and reliable set of biomarkers. Evaluation of Stabl on synthetic datasets and four independent clinical studies demonstrates improved biomarker sparsity and reliability compared to commonly used SRMs at similar predictive performance. Stabl readily extends to double- and triple-omics integration tasks and identifies a sparser and more reliable set of biomarkers than those selected by state-of-the-art early- and late-fusion SRMs, thereby facilitating the biological interpretation and clinical translation of complex multi-omic predictive models. The complete package for Stabl is available online at https://github.com/gregbellan/Stabl.

## Introduction

High-content omic technologies, such as transcriptomics, metabolomics, or cytometric immunoassays, are increasingly employed in biomarker discovery studies.^1,2^ The ability to measure thousands of molecular features in each biological specimen provides unprecedented opportunities for development of precision medicine tools across the spectrum of health and disease. Omic technologies have also dictated a shift in statistical analysis of biological data. The traditional univariate statistical framework is maladapted to large omic datasets characterized by a high number of molecular features *p* relative to the number of samples *n*. The *p* ≫ *n* scenario drastically reduces the statistical power of univariate analyses, a problem that cannot be easily overcome by increasing the value of *n* due to cost or sample availability constraints.^3,4^

Statistical analysis in biomarker discovery research comprises three related yet distinct tasks, all of which are necessary for translation into clinical use and impacted by the *p* ≫ *n* problem: 1) prediction of the clinical endpoint via identification of a multivariate model with high predictive performance (*predictivity*), 2) selection of a limited number of features as candidate clinical biomarkers (*sparsity*), and 3) confidence that the selected features are among the true set of features (i.e., truly related to the outcome, *reliability*).

Several machine-learning methods, including sparsity-promoting regularization methods (SRMs), such as least absolute shrinkage and selection operator (Lasso)^5^ or elastic net (EN),^6^ provide predictive modeling frameworks adapted to *p* ≫ *n* omic datasets, but the selection of a sparse and reliable set of candidate biomarkers remains an important challenge. Most rely on an L1-regularization to limit the number of features used in the final model. However, the learning phase of the model is often performed on a limited number of samples, such that small perturbations in the training data can yield wide differences in the features selected in the predictive model.^7–9^ This undermines confidence in the features selected, as current SRMs do not provide objective metrics to determine whether these features are truly related to the outcome. This inherent limitation of SRMs can result in poor sparsity and reliability, thereby hindering the biological interpretation and clinical significance of the predictive model. As such, few omic biomarker discovery studies progress to further clinical development phases.^1–4,10,11^

High-dimensional feature selection methods such as stability selection (SS) or Model-X knockoff improve reliability by controlling for false discoveries in the selected set of features.^12,13^ However, in these methods, the threshold for feature selection or the target false discovery rate (FDR) are defined a priori, which uncouples the feature selection from the multivariate modeling process. Without prior knowledge on the data, these methods can lead to suboptimal feature selection, requiring multiple iterations to identify a desired threshold. This limitation also precludes optimal integration of multiple omic datasets into a unique predictive model, as a single fixed selection threshold may not be suited to the specificities of each dataset.

Here we introduce Stabl, a supervised machine learning framework that bridges the gap between multivariate predictive modeling of high-dimensional omic data and the sparsity and reliability requirements of an effective biomarker discovery process. Stabl combines the injection of knockoff-modeled noise or random permutations into the original data, a data-driven signal-to-noise threshold, and integration of selected features into a predictive model. Systematic benchmarking of Stabl against Lasso, EN, and SS using synthetic datasets, three existing real-world omic datasets, and a newly generated multi-omic clinical dataset demonstrates that Stabl overcomes the shortcomings of state-of-the-art SRMs: Stabl yields highly reliable and sparse predictive models while identifying biologically plausible features amenable to further development into diagnostic or prognostic precision medicine assays.

The complete package for Stabl is available online at https://github.com/gregbellan/Stabl.

## Results

### Selection of reliable predictive features using estimated false discovery proportion (FDP)

**Fig. 1 |:**
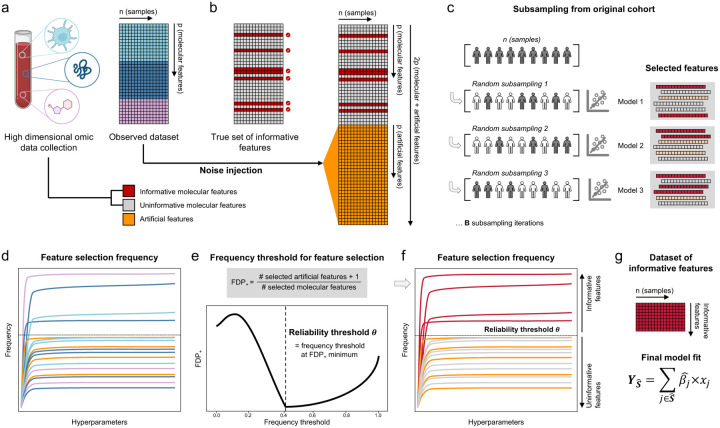
Overview of the Stabl algorithm. **a.** An original dataset of size *n* × *p* is obtained from measurement of *p* molecular features in each one of *n* samples. **b.** Among the observed features, some are informative (related to the outcome, red), and others are uninformative (unrelated to the outcome, grey). *p* artificial features (orange), all uninformative by construction, are injected into the original dataset to obtain a new dataset of size *n* × *2p*. **c.** *B* sub-sample iterations are performed from the original cohort of size *n*. At each iteration *k*, Lasso models varying in their regularization parameter *λ* are fitted on the subsample, which results in a different set of selected features for each iteration. **d.** In total, for a given *λ*, *B* sets or selected features are generated. The proportion of sets in which feature *i* is present defines the feature selection frequency *f*_!_(*λ*). Plotting*f*_!_(*λ*) against 1/*λ* yields a stability path graph. Features whose maximum frequency is above a frequency threshold (*t*) are selected in the final model. **e.** Stabl uses the reliability threshold (*θ*), obtained by computing the minimum to the false discovery proportion surrogate (FDP+, see methods). **f,g.** The set of features with a selection frequency larger than *θ* (i.e, reliable features) is included in a final predictive model.

When applied to a single cohort drawn at random from the population, SRMs will select informative features (i.e., truly related to the outcome) with a higher probability, on average, than uninformative features (i.e., unrelated to the outcome).^5,12^ However, as uninformative features typically outnumber informative features in high-dimensional omic datasets,^1,2,11^ the fit of an SRM model on a single cohort can lead to selection of many uninformative features despite a low probability of selection.^12,14^ To address this issue, Stabl implements the following strategy (Fig. 1 and methods):

1. Stabl fits SRM models (e.g., Lasso or EN) on subsamples of the data using a procedure similar to SS.^12^ Subsampling mimics the availability of multiple random cohorts and estimates each feature’s frequency of selection across all iterations. However, this procedure does not provide an optimal frequency threshold to discriminate between informative and uninformative features objectively.
2. To define the optimal frequency threshold, Stabl creates artificial features unrelated to the outcome (noise injection) via random permutations^1–3^ or knockoff sampling,^13,15,16^ which we assume behave similarly to uninformative features in the original dataset^17^ (see theoretical guarantees in methods). The artificial features are used to construct a surrogate of the false discovery proportion (FDP_+_). We define the “reliability threshold”, *θ*, as the frequency threshold yielding the minimum FDP_+_ across all possible thresholds. This method for determining *θ* is objective, in that it minimizes a proxy for the FDP. It is also data-driven, as it is tailored to individual omic datasets.

As a result, Stabl provides a unifying procedure that selects features above the reliability threshold while building a multivariate predictive model. Stabl is amenable to classification and regression tasks and extends to integration of multiple datasets of different dimensions and from different omic modalities.

### Stabl improves sparsity and reliability while maintaining predictivity: synthetic modeling

**Fig. 2 |:**
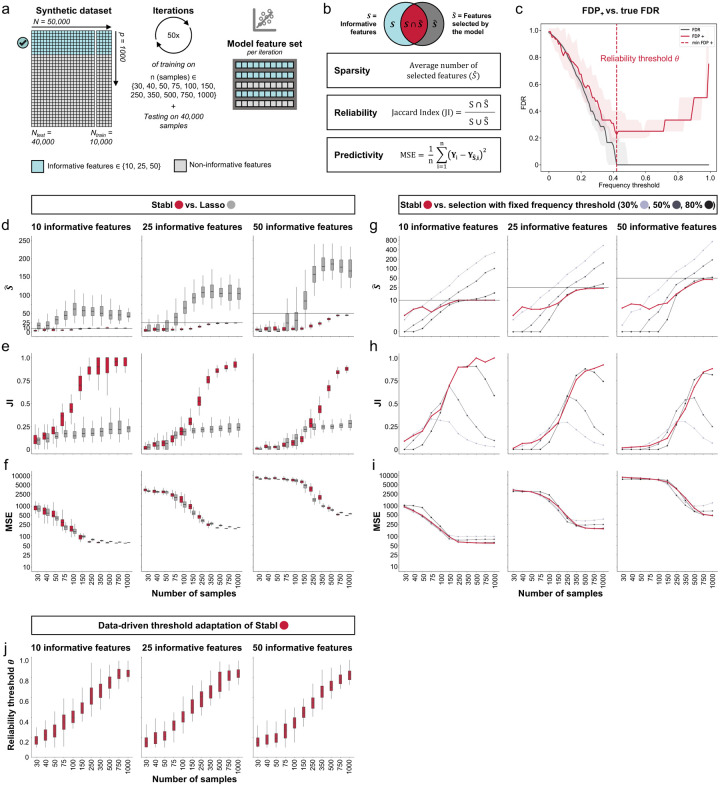
Synthetic dataset benchmarking. **a.** A synthetic dataset consisting of N = 50,000 samples × p = 1,000 features was generated. Some features are correlated with the outcome (informative features, light blue), while the others are not (uninformative features, grey). Forty thousand samples are held out for validation. Out of the remaining 10,000, 50 sets ranging of sample sizes n ranging from 30 to 1,000 are drawn randomly. **c.** Three metrics are used to evaluate performance: *sparsity* (average number of selected features compared to the number of informative features), *reliability* (Jaccard Index, JI, comparing the true set of informative features to the selected feature set), and *predictivity* (mean squared error, MSE). **c.** The surrogate for the false discovery proportion (FDP+, red line) and the experimental false discovery rate (FDR, dotted line) are shown as a function of the frequency threshold. An example is shown for n = 150 samples and 25 informative features (all other conditions are shown in Fig. S1). The FDP+ estimate approaches the experimental FDR around the reliability threshold, *θ*. **d-f**. Sparsity (**d**), reliability (JI, **e**), and predictivity performances (MSE, **f**) of Stabl (red box plots) and least absolute shrinkage and selection operator (Lasso, grey box plots) as a function of the number of samples (n, x-axis) for 10 (left panels), 25 (middle panels), or 50 (right panels) informative features. **g-i.** Sparsity (**g**), reliability (**h**), and predictivity (**i**) performances of models built using a data-driven reliability threshold *θ* (Stabl, red lines) or a fixed frequency threshold (i.e., SS) of 30% (light grey lines), 50% (Lasso, dark grey lines), or 80% (black lines). The feature set selected by Stabl remains closer in number (sparsity) and composition (reliability) to the true set of informative features, while achieving a superior or comparable predictive performance to models built using a fixed threshold. **j.** The reliability threshold chosen by Stabl is shown as a function of the sample size (n, x-axis) for 10 (left panel), 25 (middle panel), or 50 (right panel) informative features. Benchmarking of Stabl against elastic net (EN) is shown in **Fig. S6**.

We benchmarked Stabl against Lasso and EN using synthetically generated training and validation datasets containing known informative and uninformative features (Fig. 2a). Simulations representative of real-world scenarios were performed, including variations in the sample size (*n*), total features (*p*), and informative features (*S*). Models were evaluated using three performance metrics (Fig. 2b):

1. *Sparsity*: the average number of features selected compared to the number of informative features.
2. *Reliability*: overlap between the features selected by the algorithm and the true set of informative features (Jaccard Index).
3. *Predictivity*: mean square error (MSE).

Before performing benchmark comparisons, we tested whether the FDP_+_ defined by Stabl experimentally controls the FDR at the reliability threshold *θ*, as the true value of the FDR is known for the synthetic dataset. We observed that FDP_+_ (*θ*) was indeed greater than the true FDR value (Fig. 2c and S1). These observations experimentally confirmed the validity of Stabl in optimizing the frequency threshold for feature selection. Furthermore, under the assumption that the uninformative features and the artificial features are interchangeable, we bound the probability that FDP exceeds a multiple of the proximity to FDP_+_ (*θ*), thus providing a theoretical validation of our experimental observations (see theroretical guarantee in methods).

Stabl was tested using a random permutation method (Fig. 2 and S2–5) or model-X knockoffs (Fig. S5) for noise generation. In each case, Stabl achieved higher sparsity compared to Lasso or EN (Fig. S6), as the number of features selected by Stabl was lower across all conditions tested and converged towards the true number of informative features (Fig. 2d). The reliability was also higher for Stabl than for Lasso or EN, such that the features selected by Stabl were closer to the true set of informative features (Fig. 2e). Meanwhile, Stabl had similar or better predictivity compared to Lasso or EN (Fig. 2f).

Further modeling experiments tested the impact of the data-driven computation of *θ* while building the multivariate model compared to SS (i.e., choosing a fixed frequency threshold a priori). Three representative frequency thresholds were evaluated: 30%, 50%, or 80% (Fig. 2g–i and S7–9). The performance of models built using a fixed frequency threshold varied greatly depending on the simulation conditions. For example, for a small sample size (*n*<75), the 30% threshold had the best sparsity and reliability. However, for a large sample size (*n*>500), the 80% threshold resulted in greater performances. In contrast, Stabl models systematically reached optimal sparsity, reliability, and predictivity performances. Further, we show that *θ* varied greatly with the sample size (Fig. 2j and S10), illustrating how Stabl adapts to datasets of different dimensions to identify an optimal frequency threshold solution.

In sum, synthetic modeling results show that Stabl achieves better sparsity and reliability compared to Lasso or EN while preserving predictivity and that the set of features chosen by Stabl is closer to the true set of informative features. The results also emphasize the advantage of the data-driven adaptation of the frequency threshold to each dataset’s unique characteristics rather than using an arbitrarily fixed threshold.

### Stabl enables effective biomarker discovery in clinical omic studies

We evaluated Stabl’s performance on four independent clinical omic datasets. Three were previously published with standard SRM analyses, while the fourth is a newly generated dataset introduced and analyzed for the first time here. Because clinical omic datasets can vary greatly with respect to dimensionality, signal-to-noise ratio, and technology-specific data preprocessing, we tested Stabl on datasets representing a range of bulk and single-cell omics technologies, including RNA sequencing (RNA-Seq), high-content proteomics (SomaLogic and Olink platforms), untargeted metabolomics, and single-cell mass cytometry.

For each dataset, Stabl was compared to Lasso and EN on single-omic data or to early fusion and late fusion on multi-omic data over 50 random repetitions using a repeated five-fold cross-validation (CV) strategy. As the true set of informative features is not known for real-world datasets, the performance metrics differed from those used for the synthetic datasets:

1. *Sparsity*: determined by the average number of features selected throughout the CV procedure.
2. *Reliability*: assessed using univariate statistics in the absence of a known true set of features.
3. *Predictivity*: the area under the receiver operator characteristic curve (AUROC) and the area under the precision-recall curve (AUPRC) for classification tasks or the MSE for regression tasks.

### Identification of sparse, reliable, and predictive candidate biomarkers from single-omic clinical datasets

**Fig. 3 |:**
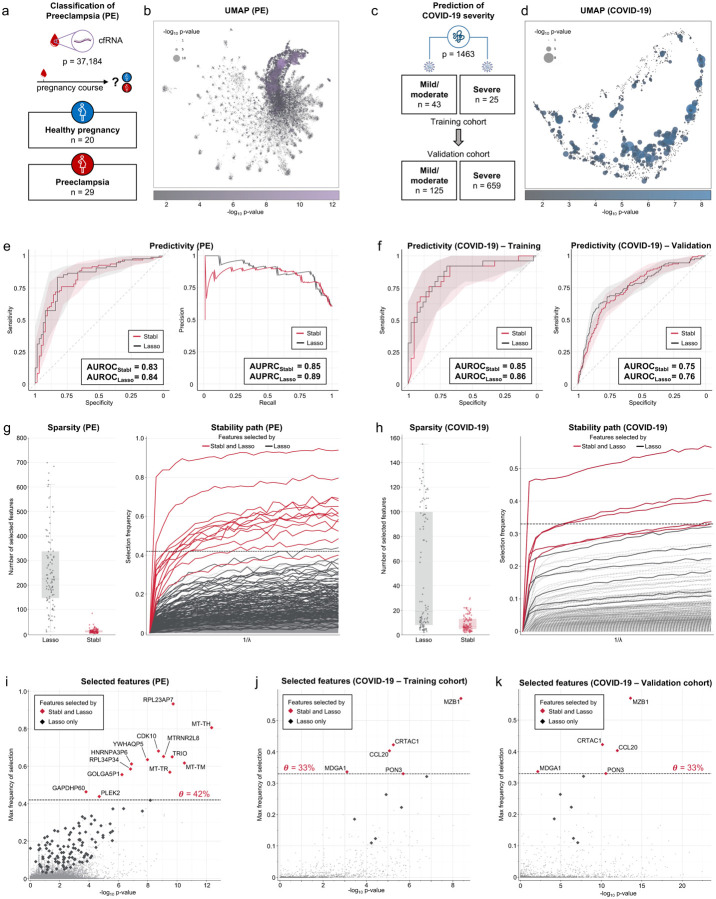
Performance of Stabl compared to Lasso on transcriptomic and proteomic data. **a**. Clinical case study 1: Classification of individuals with normotensive pregnancy or preeclampsia (PE) from the analysis of circulating cell-free RNA (cfRNA) sequencing data. Number of samples (n) and features (p) are indicated. **b.** UMAP visualization of the cfRNA transcriptomic features, node size and color are proportional to the strength of the association with the outcome calculated as the p-value in a univariate Mann-Whitney test using a −log10 scale. **c**. Clinical case study 2: Classification of mild vs. severe COVID-19 in two independent patient cohorts from the analysis of plasma proteomic data (Olink). **d.** UMAP visualization of the proteomic data. Node characteristics as in (**b**). **e.** Predictivity performances of Stabl and Lasso for the PE datasets. *AUROC*_*Stabl*_ = *0.83 [0.76, 0.90]*, *AUROC*_*Lasso*_ = *0.84 [0.78, 0.90] (p-value = 0.28, Bootstrap test); AUPRC*_*Stabl*_ = *0.85 [0.77, 0.93]*, *AUPRC*_*Lasso*_ = *0.89 [0.83, 0.94] (p-value = 0.18)* **f.** AUROC comparing predictive performance of Stabl and Lasso on training (left panel) and validation (right panel) cohorts for the COVID-19 dataset. Training: *AUROC*_*Stabl*_ = *0.85 [0.74, 0.94]*, *AUROC*_*Lasso*_ = *0.86 [0.75, 0.94] (p-value = 0.37)*. Validation: *AUROC*_*Stabl*_ = *0.75 [0.71, 0.79]*, *AUROC*_*Lasso*_ = *0.76 [0.71, 0.81] (p-value = 0.44)*. AUPRC are shown in Fig. S12. **g-h.** Left panels. Sparsity performances for the PE (**g**, number of features selected across cross-validation iterations, *median*_*Stabl*_ = *11.0, IQR = [7.8,16.0], median*_*Lasso*_ = *225.5, IQR = [147.5,337.5], p-value* < *1e-16*) and COVID-19 (**h,** *median*_*Stabl*_ = *7.0, IQR = [4.8,13.0], median*_*Lasso*_ = *19.0, IQR = [8.0,100.0], p-value = 4e-10)* datasets. Right panels. Stability path graphs showing the regularization parameter against the selection frequency. The reliability threshold (*θ*), is indicated (dotted line) **i-k**. Volcano plots depicting the reliability performances of Stabl and Lasso for the PE (**i**), COVID-19 training (**j**) and COVID-19 validation (**k**) datasets. The maximum frequency of selection of each feature is plotted against the −log10 p-value using a univariate Mann-Whitney test. Features selected by Stabl/Lasso only are colored in red/black respectively. Features selected by Stabl are labeled. PE: *mean* −*log10(p-value)*_*Stabl*_ = *8.2; mean* −*log10(p-value)*_*Lasso*_ = *3.3*. COVID-19 training: *mean* −*log10(p-value)*_*Stabl*_ = *5.5; mean* −*log10(p*-*value)*_*Lasso*_ = *5.2*. COVID-19 validation: *mean* −*log(p-value)*_*Stabl*_ = *9.7; mean* −*log10(p-value)*_*Lasso*_ = *7.8*. Benchmarking of Stabl against elastic net (EN) is shown in **Fig. S11**.

Stabl was first applied to two single-omic clinical datasets featuring a robust biological signal with significant diagnostic potential. The first example is a large-scale plasma cell-free RNA dataset (*p = 37,184* cfRNA features) isolated from pregnant patients with the aim of classifying normotensive or preeclamptic (PE) pregnancies (Fig. 3a,b).^18,19^ The second example is a high-plex proteomic dataset (*p = 1,463* proteomic features, Olink) collected from two independent cohorts (a training and a validation cohort) of SARS-CoV-2-positive patients to classify COVID-19 disease severity (Fig. 3c,d).^20,21^ In these two examples, although both Lasso and EN models achieved very good predictive performance (*AUROC* > *0.80*, Fig. 3, S11–12), the lack of sparsity or reliability hindered the identification of a manageable number of candidate biomarkers, necessitating additional feature selection methods that were decoupled from the predictive modeling process.^18–21^

Consistent with the results obtained using synthetic data, Stabl achieved comparable predictivity to Lasso (Fig. 3e,f) and EN (Fig. S11a,b) when applied to the single-omic datasets. However, Stabl identified sparser models. For the PE dataset, the average number of features selected by Stabl was reduced over 20-fold compared to Lasso (Fig. 3g) or EN (Fig. S11c) respectively. For the classification of patients with mild or severe COVID-19, the number of features selected by Stabl was reduced by a factor of 2.7 compared to Lasso (Fig. 3h) and 4.5 compared to EN (Fig. S11d).

Stabl’s reliability performance was also improved compared to Lasso and EN. The univariate p-values (Mann-Whitney test) for the features selected by Stabl were lower than for those selected by Lasso (Fig. 3i,j) or EN (Fig. S11e,f). Independent evaluation of the COVID-19 validation dataset confirmed these results (Table S1): 100% of features selected by Stabl passed a 5% FDR threshold (Benjamini-Hochberg correction) on the COVID-19 validation dataset (*mean* −*log[p-value] = 9.0*), compared to 91% for Lasso (*mean* −*log[p-value] = 6.7*, Fig. 3k) and 85% for EN (*mean* −*log[p-value] = 6.2*, Fig. S11g).

Stabl was also compared to SS using 30%, 50%, and 80% fixed frequency thresholds (Table S2). Consistent with the synthetic modeling analyses, the predictivity and sparsity performances of SS varied greatly with the choice of threshold, while Stabl provided a solution that optimized sparsity while maintaining predictive performance. For example, using SS with a 30% compared to a 50% threshold resulted in a 42% decrease in predictivity for the COVID-19 dataset (*AUROC*_*30%*_ = *0.85 vs*. *AUROC*_*50%*_ = *0.49*), with a model selecting no features. Conversely, for the PE dataset, fixing the frequency threshold at 30% *vs*. 50% resulted in a 5.3 fold improvement in sparsity with only a 6% decrease in predictivity (*AUROC*_*30%*_ = *0.83 vs*. *AUROC*_*50%*_ = *0.78*).

Identification of fewer and more reliable features using Stabl facilitated the biomarker discovery process, pinpointing the most informative biological features associated with the clinical outcome. For example, three out of thirteen (23%) cfRNA features (CDK10,^22^ TRIO,^23^ and PLEK2^24^) selected by the final Stabl PE model encoded proteins with fundamental cellular function, providing biologically-plausible biomarker candidates. Other features were non-coding RNAs or pseudogenes, with yet unknown biological function (Table S3). For the COVID-19 dataset, several features identified by Stabl echoed key pathobiological mechanisms of the host inflammatory response to COVID-19. For example, CCL20 is a known element of the COVID-19 cytokine storm,^25,26^ CRTAC1 is a newly identified marker of lung function,^27–29^ PON3 is a known biomarker decreased during acute COVID-19 infection,^30^ and MZB1 is a protein associated with high neutralization antibody titers after COVID-19 infection (Fig. 3j).^20^ The Stabl model also selected MDGA1, a previously unknown biomarker candidate of COVID-19 severity (Table S4).

Together, the results show that Stabl improves the reliability and sparsity of biomarker discovery in two single-omic datasets of widely different dimensionality while maintaining predictivity performance.

### Stabl successfully extends to multi-omic data integration

We extended the assessment of Stabl to complex clinical datasets combining multiple omic technologies. In this case, the algorithm first selects a reliable set of features at the single-omic level, then integrates the features selected for each omic dataset in a final learner algorithm, such as linear or logistic regression.

**Fig. 4 |:**
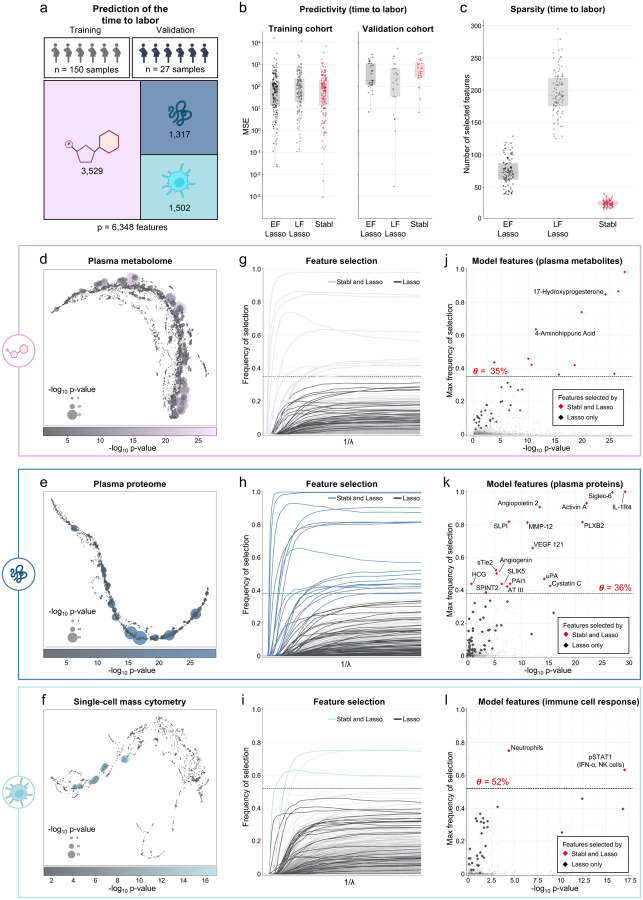
Stabl’s performances on a triple-omic data integration task. **a.** Clinical case study 3. Prediction of the time to labor from the longitudinal assessment of plasma proteomic (Olink), metabolomic (untargeted mass spectrometry), and single-cell mass cytometry datasets in two independent longitudinal cohorts of pregnant individuals. **b.** Predictivity performances (MSE, median, and IQR) for early-fusion (EF), late-fusion (LF) Lasso and Stabl, on the training (left panel) and validation (right panel) cohorts. **c.** Sparsity performances (number of features selected across cross-validation iterations, *median*_*Stabl*_ = *25.0, IQR = [22.0,29.0], median*_*EF*_ = *73.0, IQR = [61.8,87.3], p-value* < *1e-16, median*_*LF*_ = *191.5, IQR = [175.8,218.8], p-value* < *1e-16*. **d-f.** UMAP visualization of the metabolomic (**d**), plasma proteomic (**e**), and single-cell mass cytometry (**f**) datasets. Node size and color are proportional to the strength of the association with the outcome. **g-i.** Stability path graphs depicting the selection of metabolomic (**g**), plasma proteomic (**h**), and single-cell mass cytometry (**i**) features by Stabl. The data-driven reliability threshold *θ* is computed for individual omic datasets and indicated by a dotted line. **j-l.** Volcano plots depicting the reliability performances of Stabl and Lasso for each independent omic data: the metabolomics (**j**), plasma proteomic (**k**), and single-cell mass cytometry (**l**) datasets. The maximum frequency of selection of each feature is plotted against the −log10 p-value using a univariate Mann-Whitney test. Features selected by Stabl/Lasso only are colored in red/black respectively. Features selected by Stabl are labeled.

We compared Stabl to early and late fusion Lasso, two commonly employed strategies for multi-omic modeling, on the prediction of a continuous outcome variable from a triple-omic dataset. The analysis leveraged a unique longitudinal biological dataset collected in independent training and validation cohorts of pregnant individuals, together with curated clinical information (Fig. 4a).^31^ The study aimed to predict the difference in days between the time of blood sample collection and spontaneous labor onset (i.e., time to labor). The study addresses an important clinical need for improved prediction of labor onset in term and preterm pregnancies as standard predictive methods are inaccurate.^32,33^

The triple-omic dataset contained a proteomic dataset (*p* = 1,317 features, Somalogic), a metabolomic dataset (*p* = 3,529 untargeted mass spectrometry features), and a single-cell mass cytometry dataset (*p* = 1,502 immune cell features, see methods). When compared to early and late fusion Lasso, Stabl estimated the time to labor with comparable predictivity (Fig. 4b training and validation cohorts), while selecting fewer and more reliable features (Fig. 4c). Importantly, Stabl calculated a different reliability threshold for each omic sublayer (*θ*[Proteomics] = 36%, *θ*[Metabolomics] = 35%, *θ*[mass cytometry] = 52%, Fig. 4g–i). On the validation dataset, available for the proteomic and mass cytometry data only, 26% of features selected by Stabl passed a 5% FDR threshold (Benjamini-Hochberg correction), compared to 4% for early fusion Lasso and 5% for late fusion Lasso, showing that Stabl selected more reliable features (Table S5). These results emphasize the advantage of the data-driven threshold, as fixing a common frequency threshold across all omic layers would have been suboptimal, risking over- or under-selecting features in each omic dataset to be integrated into the final predictive model.

From a biological standpoint, Stabl streamlined the interpretation of our prior multivariate analyses,^31^ honing in on sentinel elements of a systemic biological signature predicting the onset of labor that could be leveraged for development of a blood-based diagnostic test. The Stabl model highlighted dynamic changes in 11 metabolomic, 17 proteomic, and two immune cell features with approaching labor (Fig. 4j–l, Table S6), including a regulated decrease in innate immune cell frequencies (e.g., neutrophils) and their responsiveness to inflammatory stimulation (e.g., pSTAT1 signaling response to IFNα in NK cells^34,35^), along with a synchronized increase in pregnancy-associated hormones (e.g., 17-Hydroxyprogesterone^36^), placental-derived (e.g., Siglec-6,^37^ Angiopoietin 2/sTie2^38^), and immune regulatory plasma proteins (e.g., IL-1R4,^39^ SLPI^40^).

### Stabl identifies promising candidate biomarkers from a newly generated multi-omic dataset

**Fig. 5 |:**
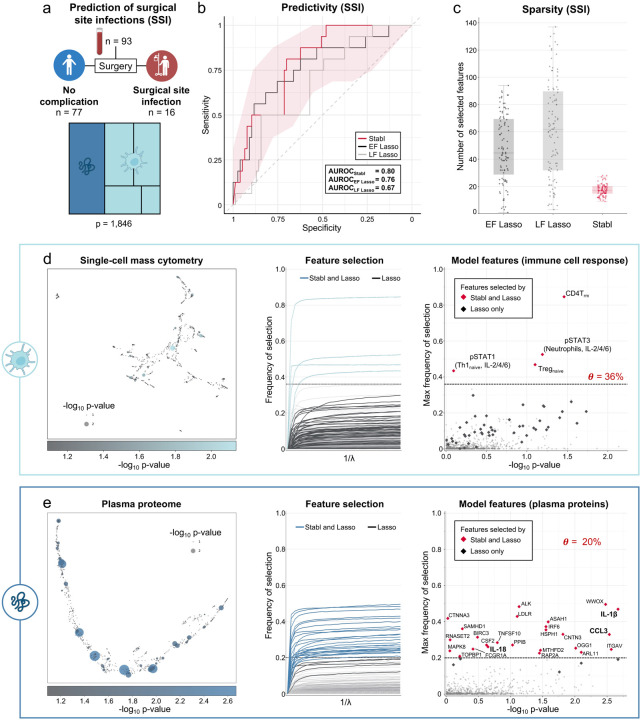
Candidate biomarker identification using Stabl for analysis of a newly generated multi-omic clinical dataset. **a.** Clinical case study 4. Prediction of postoperative surgical site infections (SSI) from the combined plasma proteomic and single cell mass cytometry assessment of pre-operative blood samples in patients undergoing abdominal surgery. **b.** Predictivity performances (AUROC) for Stabl, early fusion (EF) and late fusion (LF) Lasso. **c.** Sparsity performances (number of features selected across cross-validation iterations, *median*_*Stabl*_ = *17.0, IQR = [15.0,20.0], median*_*EF*_ = *44.5, IQR = [29.0,69.3], p-value* < *1e-16, median*_*LF*_ = *62.0, IQR = [32.0,89.5], p-value* < *1e-16*. **d-e.** UMAP (left panel), stability paths (middle panel), and volcano plots (right panels) visualization of the single-cell mass cytometry (**d**) and plasma proteomics (**e**) datasets. The data-driven reliability threshold *θ* is computed for individual omic datasets and indicated by a dotted line on the volcano plots.

Application of Stabl to the three existing omic datasets demonstrated the algorithm’s performance in the context of biomarker discovery studies with a known biological signal. To complete its systematic evaluation, Stabl was applied to our newly generated multi-omic clinical study performing an unbiased biomarker discovery task. The aim of the study was to develop a model to predict which patients will develop a postoperative surgical site infection (SSI) from analysis of pre-operative blood samples (Fig. 5a). A cohort of 274 patients undergoing major abdominal surgery were enrolled and preoperative blood samples were collected. Using a matched, nested case-control design, 93 patients were selected from the larger cohort to minimize the effect of clinical or demographic confounders on identified predictive models (Table S7). These samples were analyzed using a combined single-cell mass cytometry (Fig. S13) and plasma proteomics (Somalogic) approach.

Stabl merged all omic datasets into a final model that accurately classified patients with and without SSI (*AUROC*_*Stabl*_ = *0.80 [0.69, 0.89]*). When compared to early and late fusion Lasso, Stabl had comparable predictive performance (Fig. 5b, S14), yet superior sparsity (Fig. 5c) and reliability performance (Fig. 5h,i). As a result of the frequency-matching procedure, there were no differences in major demographic and clinical variables between the two patient groups, suggesting that model predictions were primarily driven by pre-operative biological differences in patients’ susceptibility to develop an SSI.

Stabl selected four mass cytometry and 25 plasma proteomic features that were combined into a biologically interpretable immune signature predictive of SSI. Examination of Stabl features revealed cell-type specific immune signaling responses associated with SSI (Fig. 5h) that resonated with circulating inflammatory mediators (Fig. 5i, Table S8). Notably, the STAT3 signaling response to IL-6 in neutrophils was increased before surgery in patients predisposed to SSI. Correspondingly, patients with SSI had elevated plasma levels of IL-1β and IL-18, two potent inducers of IL-6 production in response to inflammatory stress.^41,42^ Other proteomic features selected by the model included CCL3, which coordinates recruitment and activation of neutrophils, and the canonical stress response protein HSPH1. These findings are consistent with previous studies showing that heightened innate immune cell responses to inflammatory stress, such as surgical trauma,^43,44^ can result in diminished defensive response to bacterial pathogens,^39^ thus increasing a patient’s susceptibility to subsequent infection.

Altogether, application of Stabl in the setting of a new biomarker discovery study provided a manageable number of candidate biomarkers of SSI, pointing at plausible biological mechanisms that can be targeted for further diagnostic or therapeutic development.

## Discussion

Stabl is a machine learning method for analysis of high-dimensional omic data designed to unify the biomarker discovery process by identifying sparse and reliable biomarker candidates within a multivariate predictive modeling framework. Application of Stabl to several real-world biomarker discovery tasks demonstrates the versatility of the algorithm across a range of omic technologies, single- and multi-omic datasets, and clinical endpoints. Results from these diverse clinical use cases emphasize the advantage of Stabl’s data-driven adaptation to the specificities of each omic dataset, which enables reliable selection of biologically interpretable biomarker candidates conducive to further clinical translation.

Stabl builds on previous methods, including Bolasso, SS, and Model-X knockoff. These methods improve reliability of sparse learning algorithms by employing a bootstrap procedure, or using artificial features.^5,12,14,16^ However, these methods rely on a fixed or user-defined frequency threshold to discriminate between informative and uninformative features. In practice, in the *p* ≫ *n* context, objective determination of the optimal frequency threshold is difficult without prior knowledge of the data, as shown by the results from our synthetic modeling. The requirement for prior knowledge impairs the capacity for predictive model building, limiting these previous methods to sole feature selection.

Stabl improves on these methods by experimentally, and, under certain assumptions, theoretically, generalizing previous false discovery rate control methods devised for model-X knockoffs and random permutation noise.^13,45,46^ Minimization of the FDP surrogate (FDP_+_) offers two main benefits. First, it expresses a trade-off between reliability and sparsity, as it is the sum of an increasing and a decreasing function of the threshold. Second, assuming exchangeability between artificial and uninformative features Stabl’s procedure guarantees a stochastic upper bound to the FDP using the reliability threshold estimate, which ensures reliability in the optimization procedure. By minimizing this function *ex-ante*, Stabl objectively defines a model fit from the procedure without requiring prior knowledge of the data.

On a synthetic dataset, we experimentally demonstrate that Stabl selects an optimal reliability threshold by minimizing the FDP_+_ and allows for improved reliability and sparsity compared to Lasso or EN at similar predictivity performance. When tested on real-world omic studies, Stabl also performed favorably compared to Lasso and EN. For each case study, the identification of a manageable number of reliable biomarkers facilitated the interpretation of the multivariate predictive model. Prior analyses of similar datasets^18,20,21,31^ required suboptimal analysis frameworks: either post-hoc analyses were performed using user-defined cut-offs for feature selection after an initial model fit, or features associated with the clinical endpoint were selected before modeling, thus risking overfitting. In contrast, Stabl embeds the discovery of reliable candidate biomarkers within the predictive modeling, alleviating the need for separate analyses.

Stabl extended readily to analysis of multi-omic datasets where a predictive model can utilize features from different biological systems. Here, Stabl offers an alternative that avoids the potential shortcomings of early and late fusion strategies. In the case of early fusion, all omic datasets are first concatenated before applying a statistical learner. This leads to optimization on all omics combined, regardless of the specific properties (e.g., dimensions, correlation structure, underlying noise) of individual omic datasets.^47–50^ In contrast, the late fusion method trains the learner on each omic data layer independently and merges the predictions into a final dataset.^19,21,31,51–53^ In this case, although a model is adapted to each omic, the resulting model does not weigh features from different omics directly against each other. Stabl analyzes each omic data layer independently and fits specific reliability thresholds before selecting the most reliable features to be merged in a final layer, thus combining the advantages of both methods. Multi-omic data integration with Stabl was particularly useful for analysis of our newly generated dataset in patients undergoing surgery. In this case, the Stabl model comprised several features that were biologically consistent across the plasma and single-cell datasets, revealing a patient-specific immune signature predictive of SSI that appears to be programmed before surgery.

Our study has several limitations. Although we demonstrate the validity and performances of Stabl experimentally and theoretically under the assumption of exchangability between artificial and uninformative features, a more general theoretical underpinning of the method will require further guarantee. In addition, our evaluation of Stabl’s performance focused on fitting Lasso and EN models as gold standard SRMs. Further development of Stabl will be needed to allow for fitting of any SRM. While Stabl is designed to simultaneously optimize reliability, sparsity, and predictivity performances, other algorithms have been developed to address each of these performance tasks individually, such as double machine learning^54^ for reliability, Boruta^55^ for sparsity, and random forest^56^ or gradient boosting^57^ for predictivity. Additional studies are required to systematically evaluate each method’s performance in comparison to, or integrated with, the Stabl statistical framework. Finally, multi-omic data integration is an active area of research. Integrating emerging algorithms such as cooperative multiview learning^58^ may further improve Stabl’s performance in multi-omic modeling tasks.

Analysis of high-dimensional omic data has transformed the biomarker discovery process but necessitates new machine learning methods to facilitate clinical translation. Stabl addresses key requirements of an effective biomarker discovery pipeline offering a unified supervised learning framework that bridges predictive modeling of clinical endpoints with selection of reliable candidate biomarkers. Stabl enabled identification of biologically plausible biomarker candidates across multiple real-world single- and multi-omic datasets, providing a robust machine learning pipeline that we believe can be generalized to all omic data.
